# Coping, Supports and Moral Injury: Spiritual Well-Being and Organizational Support Are Associated with Reduced Moral Injury in Canadian Healthcare Providers during the COVID-19 Pandemic

**DOI:** 10.3390/ijerph20196812

**Published:** 2023-09-23

**Authors:** Andrea M. D’Alessandro-Lowe, Mauda Karram, Kim Ritchie, Andrea Brown, Heather Millman, Emily Sullo, Yuanxin Xue, Mina Pichtikova, Hugo Schielke, Ann Malain, Charlene O’Connor, Ruth Lanius, Randi E. McCabe, Margaret C. McKinnon

**Affiliations:** 1 Department of Psychology Neuroscience and Behaviour, McMaster University, Hamilton, ON L8S 4L6, Canada; dalesa1@mcmaster.ca; 2 Homewood Research Institute, Guelph, ON N13 6K9, Canada; 3 Department of Psychiatry and Behavioural Neurosciences, McMaster University, Hamilton, ON L9C 0E3, Canada; 4 Trent/Fleming School of Nursing, Trent University, Peterborough, ON K9L 0G2, Canada; 5 Temetry Faculty of Medicine, University of Toronto, Toronto, ON M5S 1A8, Canada; 6 Department of Applied Psychology and Human Development, University of Toronto, Toronto, ON M5S 1V6, Canada; 7 Homewood Health Centre, Guelph, ON NIE 6K9, Canada; 8 Lawson Health Research Institute, University of Western Ontario, London, ON N6C 2R5, Canada; 9 St. Joseph’s Healthcare Hamilton, Hamilton, ON L8N 4A6, Canada

**Keywords:** moral injury, healthcare providers, coping, supports, COVID-19, spirituality, organizational support

## Abstract

Healthcare providers (HCPs) have described the onset of shame- and trust-violation-related moral injuries (MI) throughout the COVID-19 pandemic. Previous research suggests that HCPs may turn to various coping methods and supports, such as spirituality/religiosity, substance use, friends/family or organizational support, to manage workplace stress. It remains unknown, however, if similar coping methods and supports are associated with MI among this population. We explored associations between MI (including the shame and trust-violation presentations individually) and coping methods and supports. Canadian HCPs completed an online survey about their mental health and experiences during the COVID-19 pandemic, including demographic indices (e.g., sex, age, mental health history) and measures of MI, organizational support, social support, spiritual well-being, self-compassion, alcohol use, cannabis use and childhood adversity. Three hierarchical multiple linear regressions were conducted to assess the associations between coping methods/supports and (i) MI, (ii) shame-related MI and (iii) trust-violation-related MI, when controlling for age, mental health history and childhood adversity. One hundred and seventy-six (*N* = 176) HCPs were included in the data analysis. Spiritual well-being and organizational support were each significantly associated with reduced total MI (*p*’s < 0.001), shame-related MI (*p* = 0.03 and *p* = 0.02, respectively) and trust-violation-related MI (*p*’s < 0.001). Notably, comparison of the standardized beta coefficients suggests that the association between trust-violation-related MI and both spiritual well-being and organizational support was more than twice as great as the associations between these variables and shame-related MI, emphasizing the importance of these supports and the trust-violation outcomes particularly. Mental health history (*p* = 0.02) and self-compassion (*p* = 0.01) were additionally related to shame-related MI only. Our findings indicate that heightened levels of spiritual well-being and organizational support were associated with reduced MI among HCPs during the COVID-19 pandemic. Rather than placing sole responsibility for mental health outcomes on HCPs individually, organizations can instead play a significant role in mitigating MI among staff by implementing evidence-informed organizational policies and interventions and by considering how supports for spiritual well-being may be implemented into existing models of care where relevant for employees.

## 1. Introduction

The COVID-19 pandemic has highlighted the widespread experience of moral injury (MI) in the healthcare community, in relation to both shame- and trust-violation-based morally injurious events encountered during this historic period [1,2,3,4,5,6]. MI describes the profound psychological, emotional, social and spiritual sequelae that individuals may experience after exposure to events where deeply held moral and ethical values are violated. Such events are known as potentially morally injurious events (PMIEs) [7,8] and may involve self- or other-perpetrated acts of commission or omission (i.e., actions or inactions) that result in the violation of one’s moral values. MI is conceptualized along two primary outcomes: shame-related outcomes (i.e., internalizing outcomes related to self-perpetrated PMIEs) and trust-violation-related outcomes (i.e., externalizing outcomes related to PMIEs perpetrated by others) [7,9].

Throughout the COVID-19 pandemic, healthcare providers (HCPs) faced widespread PMIEs, including witnessing and having to provide sub-optimal patient care due to a lack of resources and staffing, making difficult time-limited decisions about who would receive potentially life-saving care and having to enforce visitor restrictions for patients at end-of-life [2,3,4]. Although PMIE exposure does not always lead to the development of MI, those HCPs who develop MI may experience a host of deleterious outcomes, including a shattered sense of identity, a negative outlook on the world and its safety, the onset of symptoms of depression, anxiety, or post-traumatic stress disorder (PTSD) and the emergence of profound emotions of shame, guilt, anger and betrayal [7,9,10]. Notably, exposure to PMIEs has also been associated with increased endorsement of past-year mental health disorder diagnosis (e.g., PTSD, social anxiety disorder, panic disorder) among other service professions, including Canadian Armed Forces (CAF) personnel [11]

The nature of healthcare service exposes HCPs to diverse occupational stressors (e.g., witnessing death and dying, interpersonal conflict, visitor violence, etc. [2,12,13,14]. A long-standing body of evidence suggests that HCPs rely upon supports and coping methods to care for their mental health related to occupational stressors. These coping strategies may include problem-focused planning, self-distraction, religious practices, substance use or seeking social support [15,16]. Throughout the COVID-19 pandemic, HCPs reported turning to friends and family for support, exercising, substance use, spiritual/religious practice, meditation and psychological well-being apps to manage the stresses associated with their occupational roles [17,18,19,20]. For example, among HCPs in Poland during the COVID-19 pandemic, Budzyńka and Moryś [21] reported that HCPs most commonly engaged in denial, substance use and the cessation of usual activities in relation to the demands of their workplace between August 2021 and March 2022. Notably, these behaviors may in turn be associated with long-term mental health deterioration [21]. Alternatively, Haor et al. [22] reported that Polish nurses primarily engaged in problem-focused coping (i.e., planning, counselling, seeking support) in response to pandemic stressors between November and December 2021, which may be associated with a reduction in burnout. Further, investing in hobbies, religious practices, exercise and leaning on social supports have been associated with reduced symptoms of depression, anxiety and burnout among HCPs over the pandemic period [23,24,25]. For example, among 256 HCPs in Mexico between August and November 2022, while “maladaptive coping strategies” were associated with an increased risk for depression, anxiety and stress, “resolution coping strategies” were associated with decreased risk for these mental health outcomes [26]. Of importance, however, definitions and specific examples of maladaptive and resolution coping strategies were not offered in their investigation [26].

At present, it remains unclear how coping strategies and supports may relate to MI among HCPs. Based on the extant literature regarding MI in military populations, as well as knowledge on the use of coping and supports among HCPs to deal with workplace stress, there is reason to suspect that social support [5,6,27,28], self-compassion [29,30], spirituality/religiosity [31,32,33] and substance use [34,35] may relate to MI among HCPs. Notably, these strategies rely upon the impetus of individual rather than organizational efforts, yet the provision of organizational supports may have an additional important effect in reducing MI among HCPs [5].

### 1.1. Social and Organizational Support

Meta-analytic evidence points towards social support following traumatic exposure as a protective factor against the development and maintenance of PTSD symptoms [27,36]. More recently, our group [5] suggested that social support may provide a buffer against the development of MI post-PMIE exposure among HCPs, including through its impact on cognitive appraisals of experience. In addition to these more commonly described supports from family, friends and colleagues, healthcare organizations may play a unique role in supporting HCPs and mitigating MI [5]. For example, in an investigation of MI and psychiatric distress among American HCPs in the early stages of the pandemic (Spring, 2020), a supportive workplace environment was associated with lower self-reported MI [6]. Relatedly, research conducted during the COVID-19 pandemic further supports the notion that increased social and organizational support are positively associated with increased well-being and reduced mental health challenges, including decreased symptoms of anxiety, depression and burnout among HCPs [28,37,38].

### 1.2. Self-Compassion

Previous work points towards self-compassion as a potential mitigator of MI. Self-compassion is described as “a way of relating to [oneself] in times of suffering that is characterized by increased kindness and reduced self-judgement, increased feelings of common humanity and decreased isolation, and greater mindfulness and less over-identification with difficult thoughts and feelings” [39]. Self-compassion is positively associated with increased resilience, life satisfaction and well-being [40]. Indeed, research examining the efficacy of a self-compassion program for HCPs found that increased self-compassion and well-being maintained at a three month follow-up were associated with significant reductions in secondary traumatic stress and burnout [41]. By contrast, self-condemnation associated with MI among military personnel appears to perpetuate feelings of guilt and shame and symptoms of depression [7,10]. These findings are best articulated in Griffin et al.’s [29] model of stress and coping surrounding MI and forgiveness, where self-forgiveness is viewed as an adaptive coping attempt in response to self-condemnation following self-perpetrated PMIEs. Accordingly, elevated levels of self-compassion may buffer, in part, against MI and trauma-related mental health challenges associated with pandemic service among HCPs. Indeed, in an investigation of the relation between PTSD symptoms, self-compassion and social support among emergency staff members in Iran during the COVID-19 pandemic, increased self-kindness, a dimension of self-compassion [42], was associated with reduced PTSD symptom endorsement [43].

### 1.3. Spiritual Well-Being

MI has been associated with spiritual/religious suffering [32], and research in military populations points toward the complex influence of spiritual well-being on MI. Among veterans with severe PTSD, for example, religious involvement was negatively associated with endorsement of MI [44]. Conversely, however, in an investigation of the role of spirituality among nurses who provided in-theatre care during deployment in Iraq or Afghanistan, Simmons et al. [45] found that, although most participants reported spirituality as a source of strength that helped them endure war-related challenges, a number of nurses reported that their spiritual/religious framework enhanced their moral suffering (e.g., needing to ask God for forgiveness repeatedly, PMIE dissonant with values relating to spirituality/religion). Notably, individual differences with regard to the impact of spirituality on MI were not explored among these deployed nurses due to idiosyncratic definitions of spirituality [45]. Here, it is possible that adherence to a strict spiritual or religious framework may result in heightened spiritual/religious distress post-PMIE exposure due to high moral expectations related to a stringent worldview [32].

Evidence during the COVID-19 pandemic suggested that spiritual well-being may indeed serve as a buffer against mental health challenges among HCPs. For example, in an investigation of the associations between religious coping and symptoms of anxiety and depression among Malaysian HCPs during the pandemic period, positive religious coping (i.e., reflecting a secure relationship to a higher power and connection to others) was associated with reduced anxiety and depression, and negative religious coping (i.e., reflecting spiritual tensions and struggles) was associated with increased anxiety and depression [23].

### 1.4. Substance Use

In line with Litz et al.’s [7] earlier suggestion that the onset of MI may see an increase in self-harming behaviors, such as alcohol and drug abuse, Battles and colleagues [35] reported that higher levels of PMIE exposure were associated with increased alcohol use among military members. Notably, recent work points towards substance use among HCPs (e.g., alcohol, cannabis) as a means of coping with occupational stress during the COVID-19 pandemic [18,19]. Indeed, while HCPs at times during the pandemic had the capacity to make intentional efforts to care for their health via long-term investment in emotional, mental and physical health, interminable stress was associated with the adoption of “quick fix” strategies, such as the enhanced use of drugs and alcohol, when they did not have the mental capacity to process their emotions [17]. As such, HCPs reported using substances to “numb out” as a means of achieving immediate comfort during the COVID-19 pandemic [17]. In this vein, Patel et al. [34] found that greater endorsement of PTSD symptoms was associated with higher alcohol use among Canadian HCPs during the COVID-19 pandemic.

### 1.5. The Present Study

An understanding of factors associated with MI is necessary to address MI among HCPs beyond the COVID-19 pandemic. Accordingly, the aim of the present study was to identify coping methods and supports associated with MI among Canadian HCPs, with particular emphasis on the shame and trust-violation components of MI. Based on the available literature on coping and supports in relation to the adverse psychological outcomes reviewed above, social and organizational support, self-compassion, spiritual well-being and substance use were considered in our investigation. Age, mental health history and childhood adversity were additionally considered in our predictive models based on evidence of a relation between these variables and MI and/or PTSD [11,46,47,48]. We hypothesized that organizational support, social support, spiritual well-being, and self-compassion would be associated with reduced endorsement of MI and substance use would be associated with increased endorsement of MI. Furthermore, we hypothesized that these variables would significantly explain the variance in MI and its subtypes to a greater extent than the contributions of age, mental health history and childhood adversity alone.

## 2. Materials and Methods

### 2.1. Procedure

This study is part of a larger study on HCPs’ mental health and experiences during the COVID-19 pandemic. HCPs from across Canada aged 18 and older who worked in a healthcare setting during the COVID-19 pandemic were invited to participate in an anonymous online survey housed on Research Electronic Data Capture (REDCap) [49,50] software between May 2022 and February 2023. This study was advertised via social media platforms and e-blasts from a convenience sample of consenting organizations across the country. Participants provided electronic informed consent. The survey took approximately 40–60 min to complete. Upon completing the survey, participants were invited to enter a draw to win a CAD 50 gift card.

### 2.2. Measures

Sociodemographic Questionnaire. The sociodemographic questionnaire included demographic (e.g., sex, gender, age, marital status, mental health history) and occupational information (e.g., healthcare field, occupational setting, years worked). Mental health history was assessed by asking participants to indicate yes (1) or no (0) as to whether they had ever been given a mental health diagnosis. Participants had the option to indicate the diagnosis if they wished. A dichotomous variable for mental health history was used in predictive models to account for the association between lifetime mental health concerns and MI.

Moral Injury Outcomes Scale (MIOS). The MIOS [51] is a self-report questionnaire assessing for MI. Participants rated 14 items on a 5-point rating scale ranging from 0 (Strongly Disagree) to 4 (Strongly Agree) based on their experience in the past month related to their worst morally distressing/injurious event endured. The MIOS has a two-factor structure: shame-related (e.g., “I am not the good person I thought I was,” “People would hate me if they really knew me”) and trust-violation-related outcomes (e.g., “I have trouble seeing goodness in others,” “I’m angry all the time”). Scores were calculated by summing the individual items. Cronbach’s alpha for internal consistency in our sample was 0.87 and 0.75 for the shame and trust-violation subscales, respectively.

Adverse Childhood Experiences Scale (ACES). The ACES [52] is a 10-item self-report assessment of childhood adversity. Participants reported if they have experienced a range of adverse experiences in childhood (e.g., physical abuse, neglect) on a categorical yes (1) or no (2) scale. Total scores were the sum of the 10 items, such that greater scores indicated greater exposure to childhood adversity.

Multidimensional Scale of Perceived Social Support (MSPSS). The MSPSS [53] is a self-report questionnaire assessing perceived support from family, friends and significant others. Participants rated their degree of agreement with 12 statements on a 7-point rating scale ranging from 1 (Very Strongly Disagree) to 7 (Very Strongly Agree). Total scores were calculated by taking the average of individual items. Cronbach’s alpha for internal consistency in our sample was 0.94.

Self-Compassion Scale (SCS). The SCS [42] is a self-report instrument assessing trait-level self-compassion. Participants rated their degree of agreement with 26 items on a 5-point scale ranging from 1 (Almost Never) to 5 (Almost Always). Total scores were calculated by taking the average of individual items. Cronbach’s alpha for internal consistency in our sample was 0.94.

Survey of Perceived Organizational Support (SPOS). The SPOS [54] is a self-report instrument assessing the degree to which employees perceive their organization values their contributions and cares for their well-being. Participants rated 16 items on a 7-point rating scale ranging from 0 (Strongly Disagree) to 6 (Strongly Agree). Total scores were calculated by summing the scores from the individual items. Cronbach’s alpha for internal consistency in our sample was 0.96.

Functional Assessment of Chronic Illness Therapy—Spiritual Well-Being (FACIT-Sp-12). The non-illness version of the FACIT-Sp-12 [55] is a self-report questionnaire assessing spiritual well-being. Participants rated their degree of agreement with 12 items on a 5-point scale ranging from 0 (not at all) to 4 (very much). The FACIT-Sp-12 is useful across religious traditions and among those individuals who consider themselves spiritual outside of religious contexts [55]. Total scores were calculated by taking the average of individual items. Cronbach’s alpha for internal consistency in our sample was 0.90.

Alcohol Use Disorder Identification Test (AUDIT). The AUDIT [56] is a self-report questionnaire screening for alcohol intake and dependence within the past 12 months. Participants reported how often they consumed alcohol, if their drinking resulted in self-injury or injury to others and if someone has ever recommended that they cut back on alcohol consumption. Participants rated their degree of agreement with 6 items pertaining to alcohol consumption on a 5-point scale ranging from 0 (Never) to 4 (Daily or Almost Daily). Total scores were calculated summing scores across the individual items. Cronbach’s alpha for internal consistency in our sample was 0.83.

Cannabis Use Disorder Identification Test (CUDIT). The CUDIT [57] is a self-report questionnaire screening for cannabis intake and dependence over the past 12 months. Participants reported how often they consumed cannabis, if their consumption resulted in hazardous situations and if they have ever thought about cutting back or stopping their use of cannabis. Participants rated their degree of agreement with 6 items pertaining to cannabis consumption on a 5-point scale ranging from 0 (Never) to 4 (Daily or Almost Daily). Total scores were calculated by summing the scores across the individual items. Cronbach’s alpha for internal consistency in our sample was 0.81.

### 2.3. Data Preparation & Analysis

Statistical Software for Social Sciences version 29 [58] was used for data analysis. Participants who consented to secondary analysis of their data and completed at least 90% of the items on each measure of interest for this study were included in data analysis (*N* = 176). Missing data were assessed and imputed using the estimation maximization procedure. Only 0.23% (43) of values pertaining to the analysis were missing. Little’s MCAR test [59] suggested that the missing values were missing at random [*X*^2^(3199) = 3280.84, *p* = 0.15]. Average scores were compared between the complete case and imputed samples (see Supplementary Materials for more information). Descriptive statistics were run to characterize the sample. Three hierarchical multiple linear regressions were constructed to determine the associations between organizational support, social support, spiritual well-being, self-compassion, alcohol use, cannabis use and (i) total MI; (ii) shame-related MI and (iii) trust-violation-related MI. Age, mental health history and childhood adversity were entered into the models as a single block to control for the impact of these variables on MI. All regression assumptions were assessed and met. An alpha level of *p* < 0.05 was used for analyses.

## 3. Results

### 3.1. Sample Characteristics

One-hundred and seventy-six (*N* = 176) HCPs were included in data analysis (Table 1; cells with less than five counts have been suppressed to protect anonymity). Participants ranged from 21 to 73 years old, with an average age of 43.3 (*SD* = 11.9). Most participants were female (91%) and registered nurses or registered practical nurses (56.8%). Almost half (45.5%) of the participants reported being diagnosed with a mental disorder in their lifetime. Descriptive statistics for all self-report scales are provided in Table 2.

### 3.2. Associations with Total Moral Injury Scores

Hierarchical multiple linear regression was run to determine the associations between coping methods/supports and MI, when controlling for age, mental health history and childhood adversity (Table 3). The full model significantly explained 49.3% of the variance (Adjusted *R*^2^ = 0.466) in MI [*F*(9, 166) = 17.95, *p* < 0.001], representing a 38% increase from the variance in MI explained by age, mental health history and childhood adversity alone [*R*^2^ Change = 0.38, *F*(6, 166) = 20.77, *p* < 0.001]. Perceived organizational support (*b* = −0.11, *p* < 0.001) and spiritual well-being (*b* = −4.78, *p* < 0.001) were each significantly associated with reduced MI. The positive association between cannabis use and MI approached significance (*b* = 0.29 *p* = 0.07).

### 3.3. Associations with Shame-Related Moral Injury

Hierarchical multiple linear regression was run to determine the associations between coping methods/supports and shame-related MI, when controlling for age, mental health history and childhood adversity (Table 4). The full model significantly explained 38% of the variance (Adjusted *R*^2^ = 0.346) in MI [*F*(9, 166) = 11.30, *p* < 0.001], representing a 24.1% increase from the variance in MI explained by age, mental health history and childhood adversity alone [*R*^2^ Change = 0.241, *F*(6, 166) = 10.75, *p* < 0.001]. Mental health history was significantly associated with shame-related MI, such that having a lifetime history of a mental health diagnosis was associated with an increase in shame-related MI (*b* = 2.06, *p* = 0.02). Self-compassion (*b* = −1.90, *p* = 0.01), spiritual well-being (*b* = −1.55, *p* = 0.03) and perceived organizational support (*b* = −0.04, *p* = 0.02) were each significantly associated with reduced shame-related MI. The positive association between cannabis use and shame-related MI approached significance (*b* = 0.19, *p* = 0.07).

### 3.4. Associations with Trust-Violation-Related Moral Injury

Hierarchical multiple linear regression was run to determine the associations between coping methods/supports and trust-violation-related MI, when controlling for age, mental health history and childhood adversity (Table 5). The full model significantly explained 45.9% of the variance (Adjusted *R*^2^ = 0.430) in MI [*F*(9, 166) = 15.64, *p* < 0.001], representing a 40.4% increase from the variance in MI explained by age, mental health history and childhood adversity alone [*R*^2^ Change = 0.404, *F*(6, 166) = 20.63, *p* < 0.001]. Perceived organizational support (*b* = −0.07, *p* < 0.001) and spiritual well-being (*b* = −3.23, *p* < 0.001) were each significantly associated with decreased trust-violation-related MI.

## 4. Discussion

The primary aim of this study was to investigate the relation between MI and coping methods and supports among Canadian HCPs during the COVID-19 pandemic. Higher levels of spiritual well-being and perceived organizational support were each associated with a reduced endorsement of MI. Cannabis use approached significance as being associated with an elevated endorsement of MI. The addition of coping methods and supports to a predictive model explained an additional 38% of the variance of MI, above the contributions of age, mental health history and childhood adversity to MI. Indeed, in the final step of the model in which coping methods/supports were added, the significant associations between age, mental health history and childhood adversity were no longer significantly associated with MI.

Our secondary aim was to elucidate associations between the coping methods and supports and shame and trust-violation presentations of MI, separately. We found that the inclusion of coping methods and supports explained an additional 24.1% variance in shame-related MI and an additional 40.4% of the variance in trust-violation-related MI, beyond the contributions of age, mental health history and childhood adversity. Notably, in the final step of the model, childhood adversity was no longer significantly associated with shame-related MI, yet mental health history maintained a significant association with increased shame-related MI. Alternatively, neither age, mental health history, nor childhood adversity were significantly associated with trust-violation-related MI in the final step of the model. Spiritual well-being and organizational support were each associated with increased endorsement of shame-related and trust-violation-related MI. Comparison of the standardized beta coefficients in our models revealed that the strength of the association between trust-violation-related MI and both spiritual well-being and organizational support was more than twice as great as the associations between these variables and shame-related MI, emphasizing the potential importance of these supports and the trust-violation outcomes of MI compared to the shame-related outcomes of MI. Higher levels of self-compassion were also significantly associated with reduced shame-related MI, but not trust-violation-related MI. Cannabis use approached significance in predicting higher levels of shame-related MI, but not trust-violation-related MI. These results suggest that the utility of self-compassion and the use of cannabis may depend on the type of PMIE exposure. Specifically, individuals exposed to self-perpetrated PMIEs, which tend to give rise to the shame-related presentation of MI, may benefit from self-compassion training and may be more likely to utilize cannabis as a coping method than those exposed to PMIEs perpetrated by others, commonly associated with the trust-violation presentation of MI [7,9,51]. Further investigation is required in this area, however, provided the limited literature addressing substance use and trust- versus shame-related MI. Additionally, the temporal relationship between cannabis use and shame-related MI was not explored in our study. As such, it is unclear whether individuals experienced a PMIE followed by shame-related MI and then turned to cannabis to cope, or if cannabis use was endorsed prior to PMIE exposure. Further research is needed to extend the results of our association study and understand the temporal relationship between substance use and shame-related MI.

Taken together, these findings deepen our understanding of factors associated with MI among HCPs by identifying the additional impact of coping methods and supports beyond demographic and life history (i.e., age, childhood adversity, mental health diagnosis) variables alone. This observation is particularly germane for trust-violation MI, in which demographic and life history factors alone explained only approximately 5.5% of the variance in MI. Critically, these findings also suggest that where exposure to workplace PMIEs may be inevitable for many HCPs, the provision of coping methods and supports appears beneficial in warding against MI. Here, emphasis on supports that enhance spiritual well-being and bolster perceived levels of organizational support may serve as key avenues to reducing MI in this vital workforce.

### 4.1. Spiritual Well-Being

In the present study, spiritual well-being was strongly associated with reduced MI among HCPs, especially with trust violation compared to the shame outcome. Notably, spiritual well-being was conceptualized through the FACIT-Sp-12 as a measure not specific to any one religious or spiritual tradition [55]. Spiritual or existential conflict are conceptualized within the core of MI (e.g., challenged core beliefs, relationships with self, others, or the sacred/transcendent), in which interventions aimed at enhancing spiritual well-being may prove, in part, effective in the aftermath of PMIE exposure [32,60]. For clients valuing spirituality, clinical interventions that consider spirituality/religiosity post-PMIE exposure may assist in bridging patients to psychotherapeutic inventions by first reconciling worldviews, resolving anger at God or god figures and fostering reconciliation to communities [32,33]. Consistent with this assumption, mental health chaplains have long been central to the mental health care model for military members, offering formal mental health supports in line with “spirituality-integrated holistic person-centered care.” This biopsychosocial–spiritual (BPSS) model expands the traditional biopsychosocial model to incorporate spirituality as part of a continuum of care (i.e., from spirituality screening tools to treatment), providing a more holistic care approach to clients valuing spirituality as central to their worldview [61]. Empirical evidence points toward the success of interventions based on the BPSS model in military personnel and veterans [32,33], palliative/end-of-life care [62] and families [63], in which the BPSS model views spirituality as a central domain of health-related quality of life, alongside physical, mental and social well-being [55]. As seen in existing military approaches, augmenting mental health care teams with a spiritual care provider may prove beneficial for some HCPs in supporting spiritual well-being and mental health post-PMIE exposure by allowing HCPs a safe space to wrestle with cognitions and affects that highly contradict with their worldviews [32,61]. As our data reveal a stronger association between spiritual well-being and trust-violation-related MI than between spiritual well-being and shame-related outcomes, this approach may be particularly relevant in cases where PMIEs involved witnessing moral violations by the actions or inactions of others (e.g., systematic or organizational-related PMIEs). Caution is warranted, however, in the application of this model, as some clients may view spirituality as of little relevance or even potentially harmful. As such, where aligned with individuals’ belief systems, formal and informal spiritual well-being may then serve as an avenue for healing feelings of grief, anger, mistrust, guilt, or shame that often accompany experiences of PMIEs [32,61]

Although our findings are consistent with previous evidence suggesting that spirituality and/or positive religious coping (e.g., having a desirable relationship with God) is associated with decreased MI, depression and anxiety, it is worth noting that prior evidence also suggests that spirituality and/or negative religious coping (e.g., belief that experiences are God’s punishment or abandonment) may exacerbate HCPs’ MI, PTSD, depression and anxiety and even reduce quality of life [23,45,64]. For example, Wang and colleagues [64] found that Chinese HCPs’ reports of spirituality were positively associated with MI and depressive and anxiety symptoms. The authors speculated that HCPs with a higher degree of religiosity may hold themselves to strict moral standards or may feel a greater degree of moral responsibility when it comes to decisions regarding the well-being of others which, when faced with resistance by system-related problems, may result in increased helplessness and associated adverse mental health outcomes [64]. Further, the relation between spirituality and mental health difficulties may be explained, in part, by the onset of guilt, lack of meaning and purpose, and spiritual and moral disintegration following exposure to PMIES [33,55,64]. Although our results point towards positive benefits of enhanced spirituality in mitigating MI among HCPS exposed to PMIEs during the COVID pandemic, we cannot bar individual differences in the expression of this benefit that remain to be identified. Further work is needed to better understand the circumstances and mechanisms through which spiritual well-being may impact MI.

Additionally, although our results demonstrate an association between spiritual well-being and MI, we are unable to make conclusions about the temporal relationship between these variables. As discussed above with cannabis use, it remains unclear whether individuals specifically turned to resources to enhance spiritual well-being after PMIE exposure, or if individuals previously strong in spiritual well-being were less impacted by MI after experienced a PMIE. Future research is needed to better understand this complex relationship.

### 4.2. Organizational Support

Consistent with previous work [6,65,66,67], higher levels of perceived organizational support were significantly associated with reduced MI in our sample of HCPs, particularly in relation to trust-violation outcomes of MI. Although organizations may assist HCPs facing PMIEs in the workplace by providing mental health resources (e.g., employee assistance programming, benefits), they must also acknowledge their unique role as a key tenant of support for their employees [5]. Cohen and Wills [68] proposed that social support may mitigate adverse psychological outcomes post-trauma exposure when there is an appropriate match between the exposure and the support received. In the healthcare context, organizations must ensure that support offered to their employees adequately responds to the PMIEs and other stressors HCPs endure in the workplace [5]. This includes first considering how the organization itself may contribute to PMIEs in the workplace and second adequately revising policies and procedures to reduce the risk of these types of stressors where possible. In a qualitative investigation of work-related PMIEs during the COVID-19 pandemic, Canadian HCPs reported working with limited resources and personal protective equipment, taking on increased workloads with decreased staffing levels, and dissonance between HCPs’ and management’s values as PMIEs [2]. In these situations, HCPs may experience MI, especially trust-violation outcomes related to witnessing the violation of moral values by an institution. Organizations can communicate their support to HCPs by creating space for collaboration in decision making, establishing a shared purpose and identity, facilitating team discussions, training supportive leaders, allowing for rotations between high and low stress areas and acknowledgement and affirmation of HCPs’ sacrifice on the frontlines [65,66,67].

Notably, our results suggest that mental health history and self-compassion relate to shame-related MI among HCPs. Specifically, whereas a lifetime history of a mental health diagnosis was associated with increased shame-related MI, increased self-compassion was associated with decreased shame-related MI in the final step of the model. This finding is in line with Hall et al.’s [69] review of MI, mental health and behavioral outcomes, in which self-compassion moderated outcomes related to PMIE exposure, including suicidality, depression, PTSD. Surprisingly, however, in the current study, neither mental health history nor self-compassion was significantly associated with the final step of the trust-violation-related MI model among HCP. Self-compassion thus appears particularly relevant in reconciling internalized outcomes related to self-perpetrated PMIEs. Here, Neff’s [42] conceptualization of self-compassion addresses forgiving one’s perceived failures and shortcomings and considering one’s suffering as part of the common human experience without harsh self-condemnation. When coping with stress, self-compassion may enable both an emotional and a problem-focused approach to coping [42], where an emotional approach to coping may create space for individuals to deal with their emotions in a psychologically adaptive way, as opposed to emotional avoidance (e.g., laughing things off as unimportant) [42] or self-condemnation. In line with this notion, Neff [42] suggested that a self-compassionate individual may be more aware of how their own actions may be maintaining or exacerbating a stressful experience, enabling them to take a more problem-focused approach to coping. Future research may build on this finding and explore the mechanisms through which self-compassion may reduce MI among HCPs.

### 4.3. Limitations

Although this research yields novel evidence of the relation between MI and coping methods/supports, interpretation of the present results must be tempered according to methodological limitations. The results of this study may not be generalizable to HCPs across Canada or globally due to the sample being composed mainly of female, Caucasian nurses in Ontario with nearly half reporting a lifetime history of at least one mental health diagnosis. Moreover, although these data were collected during the COVID-19 pandemic, the present results may not be specific to experiences during the pandemic as the instrument used (MIOS) did not specify a time frame for participants to select a PMIE. Additionally, although the coping methods and supports used in our analyses map on well to the types of supports and coping methods reported by HCPs during the pandemic, we did not include an exhaustive list of any and all supports and coping methods in our models (e.g., food, television) that may also relate to MI. Further, clinically relevant differences in scores on the SPOS and FACIT-Sp were not accessible, thus rending it unclear as to what extent our statistically significant results indicate meaningful clinical or psychological differences. In addition, our work is correlational in nature and cannot comment on the potentially complex, temporal relationships between coping/supports and MI. Finally, a lack of consensus on what constitutes MI and PMIEs in the healthcare context remains a limitation for all work in this area. Nonetheless, the present study contributes to a growing body of knowledge surrounding MI among HCPs during the COVID-19 pandemic and points towards coping/supports associated with MI, which may be useful targets in interventions.

## 5. Conclusions

Overall, our findings suggest that higher levels of spiritual well-being and perceived organizational support were associated with reduced MI among HCPs during the COVID-19 pandemic, a relation strongest for trust-violation-related MI. This knowledge should inform discussions on how to best support HCPs beyond the pandemic period. Instead of placing sole responsibility on HCPs for their mental health, organizations have a significant role to play in mitigating MI among staff by offering appropriate supports and attitudes and by considering how, for some HCPs, spiritual well-being may be implemented into existing models of care for staff. Future research is needed to better understand the potentially complex temporal relationships between coping/supports and MI and to develop evidence-informed resources for mitigating MI among HCPs beyond the pandemic period.

## Figures and Tables

**Table 1:** Sociodemographic Information.

Variable	Level	Frequency	Percentage
Sex			
	Male	16	9.1
	Female	160	90.9
Gender			
	Female	157	89.2
	Male	17	9.7
	Other	<5	-
Racial Identity			
	African/Caribbean	<5	-
	Caucasian	149	84.7
	East Asian	<5	-
	Indigenous	<5	-
	Latin American	<5	-
	Southeast Asian	<5	-
	Mixed	<5	-
	Other	8	4.5
Province			
	Alberta	15	8.5
	British Columbia	18	10.2
	Manitoba	13	7.4
	New Brunswick	<5	-
	Newfoundland and Labrador	<5	-
	Northwest Territories	<5	-
	Nova Scotia	7	4.0
	Ontario	110	62.5
	Prince Edward Island	<5	-
	Quebec	<5	-
	Saskatchewan	<5	-
	Yukon	<5	-
Profession			
	Registered Nurse/Registered Practical Nurse	100	56.8
	Medical Physician	7	4.0
	Respiratory Therapist	<5	-
	Personal Support Worker	9	5.1
	Occupational Therapist	<5	-
	Physiotherapist	<5	-
	Social Worker	15	8.5
	Recreational Therapist	<5	-
	Psychologist	<5	-
	Other	38	20.5
Occupational Setting			
	Acute Care Hospital	72	40.9
	Primary Care	10	5.7
	Mental Health Hospital	7	4.0
	Rehabilitation Hospital	<5	-
	Long-Term Care or Retirement	47	26.7
	Community or Home Care	13	7.4
	Public Health	6	3.4
	Other	20	11.4
Years Worked			
	0 to 5 Years	54	30.7
	6 to 10 Years	33	18.8
	11 to 15 Years	31	17.6
	16 to 20 Years	17	9.7
	21 to 25 Years	10	5.7
	26 to 30 Years	8	4.5
	31+ Years	23	13.1
Mental Health History			
	Suicidality	12	6.8
	Major Depressive Disorder	35	19.9
	Persistent Depressive Disorder	15	8.5
	Bipolar I Disorder	5	2.8
	Bipolar II Disorder	<5	-
	Panic Disorder	14	8.0
	Agoraphobia	<5	-
	Social Anxiety Disorder	15	40.9
	Generalized Anxiety Disorder	36	20.5
	Obsessive Compulsive Disorder	36	20.5
	Post-Traumatic Stress Disorder	37	21.0
	Acute Stress Disorder	16	9.1
	Alcohol Use Disorder	<5	-
	Substance Use Disorder	<5	-
	Anorexia Nervosa	5	2.8
	Bulimia Nervosa	<5	-
	Binge Eating Disorder	<5	-
	Specific Phobia	<5	-
	Personality Disorder	<5	-
	Other	18	10.2

**Table 2:** Descriptive Statistics for Self-Report Scales.

Construct	Scale	Median	*M*	*SD*
Moral Injury	MIOS	23.00	22.35	10.10
Shame	MIOS	9.00	9.08	6.21
Trust Violation	MIOS	14.00	13.27	5.28
Childhood Adversity	ACES	2.00	2.49	2.33
Organizational Support	SPOS	49.50	52.82	25.02
Social Support	MPSS	5.38	5.18	1.32
Spiritualty/Religiosity	FACIT-Sp-12	1.92	1.98	0.83
Self-Compassion	SCS	2.62	2.70	0.70
Alcohol Use	AUDIT	2.00	3.97	4.83
Cannabis Use	CUDIT	0.00	1.85	3.92

**Table 3:** Hierarchical Multiple Linear Regression of Moral Injury.

		Unstandardized Coefficients					95% Confidence Interval	
		*b*	*SE*(*b*)	β	*t*	*p*	Lower Bound	Upper Bound
1	Constant	24.65	2.90		8.51	<0.001 **	18.93	30.37
	Age	−0.14	0.06	−0.16	−2.18	0.03 *	−0.26	−0.01
	Mental Health History	4.03	1.56	0.20	2.58	0.01 **	0.95	7.11
	Childhood Adversity	0.68	0.33	0.16	2.04	0.04 *	0.02	1.34
2	Constant	39.64	3.73		10.63	<0.001 **	32.28	47.01
	Age	0.02	0.05	0.02	0.30	0.77	−0.09	0.12
	Mental Health History	1.76	1.25	0.09	1.40	0.16	−0.72	4.23
	Childhood Adversity	0.34	0.27	0.08	1.26	0.21	−0.19	0.87
	Organizational Support	−0.11	0.02	−0.27	−4.47	<0.001 **	−0.16	−0.06
	Social Support	−0.25	0.50	−0.03	−0.50	0.62	−1.24	0.74
	Spiritual well-being	−4.78	1.05	−0.39	−4.53	<0.001 **	−6.86	−2.70
	Self-Compassion	−1.36	1.12	−0.09	−1.22	0.23	−3.57	0.85
	Alcohol Use	−0.00	0.12	−0.00	−0.02	0.99	−0.24	0.24
	Cannabis Use	0.29	0.16	0.11	1.83	0.07	−0.02	0.59

* *p* < 0.05; ** *p* < 0.01.

**Table 4:** Hierarchical Multiple Linear Regression of Shame-Related Moral Injury.

		Unstandardized Coefficients					95% Confidence Interval	
		*b*	*SE*(*b*)	β	*t*	*p*	Lower Bound	Upper Bound
1	Constant	8.80	1.76		5.01	<0.001 **	5.33	12.26
	Age	−0.05	0.04	−0.10	−1.41	0.16	−0.13	0.02
	Mental Health History	3.35	0.95	0.27	3.55	<001 **	1.49	5.22
	Childhood Adversity	0.42	0.20	0.16	2.06	0.04 *	0.02	0.82
2	Constant	17.95	2.54		7.07	<0.001 **	12.94	22.96
	Age	0.03	0.03	0.06	0.85	0.40	−0.04	0.10
	Mental Health History	2.06	0.85	0.17	2.41	0.02 *	0.37	3.74
	Childhood Adversity	0.19	0.18	0.07	1.01	0.31	−0.18	0.55
	Organizational Support	−0.04	0.02	−0.16	−2.38	0.02 *	−0.07	−0.01
	Social Support	−0.31	0.34	−0.07	−0.92	0.36	−0.98	0.36
	Spiritual well-being	−1.55	0.72	−0.21	−2.17	0.03 *	−2.97	−0.14
	Self-Compassion	−1.90	0.76	−0.21	−2.49	0.01 **	−3.40	−0.40
	Alcohol Use	−0.00	0.08	−0.00	−0.03	0.97	−0.17	0.16
	Cannabis Use	0.19	0.11	0.12	1.80	0.07	−0.02	0.40

* *p* < 0.05, ** *p* < 0.01.

**Table 5:** Hierarchical Multiple Linear Regression of Trust-Violation-Related Moral Injury.

		Unstandardized Coefficients					95% Confidence Interval	
		*b*	*SE*(*b*)	β	*t*	*p*	Lower Bound	Upper Bound
1	Constant	15.85	1.56		10.13	<0.001 **	12.76	18.94
	Age	−0.08	0.03	−0.18	−2.46	0.02 *	−0.15	−0.02
	Mental Health History	0.68	0.84	0.06	0.80	0.42	−0.98	2.34
	Childhood Adversity	0.26	0.18	0.12	1.46	0.15	−0.09	0.62
2	Constant	21.69	2.02		10.76	<0.001 **	17.71	25.67
	Age	−0.01	0.03	−0.03	−0.52	0.60	−0.07	0.04
	Mental Health History	−0.30	0.68	−0.03	−0.44	0.66	−1.63	1.04
	Childhood Adversity	0.16	0.15	0.07	1.06	0.29	−0.13	0.44
	Organizational Support	−0.07	0.01	−0.33	−5.28	<0.001 **	−0.09	−0.04
	Social Support	0.06	0.27	0.02	0.23	0.82	−0.47	0.60
	Spiritual well-being	−3.23	0.57	−0.51	−5.66	<0.001 **	−4.35	−2.10
	Self-Compassion	0.54	0.61	0.07	0.89	0.38	−0.66	1.73
	Alcohol Use	0.00	0.07	0.00	0.01	0.99	−0.13	0.13
	Cannabis Use	0.09	0.08	0.07	1.11	0.27	−0.07	0.26

* *p* < 0.05, ** *p* < 0.01.

## Data Availability

The data used in this study come from the McKinnon Trauma and Recovery Research Unit at McMaster University. All interested researchers may apply for access to these data through online application subject to review by the Data Access Committee, ethics approval and signing of a data sharing agreement. Data are provided only once a data sharing agreement is in place between McMaster University (the custodian of the data) and the researchers’ institution. For more information about data access please contact https://www.thetraumaandrecoverylab.com/contact, accessed on 8 September 2023.

## Supplementary Materials

The following supporting information can be downloaded at: https://www.mdpi.com/article/10.3390/ijerph20196812/s1, Figure S1: Summary of Missing Data; Table S1: Average Scores and Standard Deviations for Complete Case Sample and Imputed Sample.
