# Supplementary material for: Coping, Supports and Moral Injury: Spiritual Well-Being and Organizational Support Are Associated with Reduced Moral Injury in Canadian Healthcare Providers during the COVID-19 Pandemic
Source: Int J Environ Res Public Health. 2023 Sep 23;20(19):6812. doi: 10.3390/ijerph20196812 (PMC10572244; doi:10.3390/ijerph20196812)
Supplement: Supplementary file 1 [file ijerph-20-06812-s001.zip › ijerph-2576056-supplementary.pdf]

# Supplementary Materials

## Data Preparation

Statistical Software for Social Sciences version 29 was used for data analysis. Participants who consented to secondary analysis of their data and completed at least 90% of the items that contribute to a total score on each measure of interest (i.e., MIOS, ACES, SPOS, MSPSS, FACIT-Sp, SCS, AUDIT, CUDIT) for this study were included in data analysis (N=176). Missing data was assessed on SPSS. The breakdown of missing data is presented in Figure S1.

**Figure S1**

*Overall Summary of Missing Values*

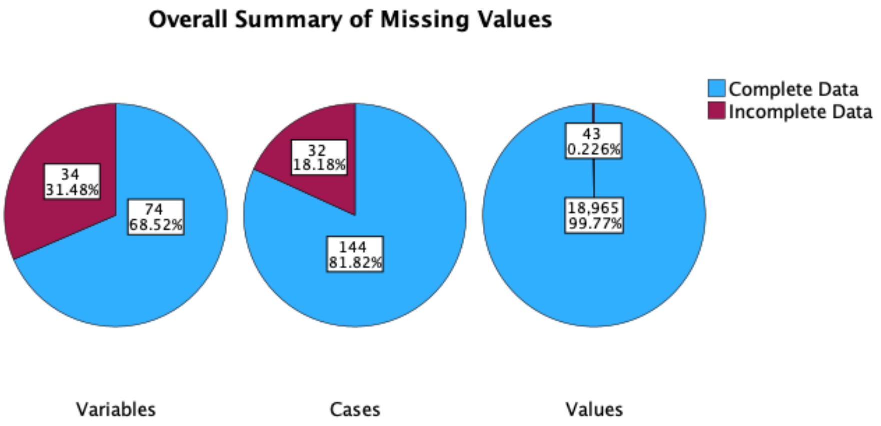


Of the 108 variables which constitute the total scores for the variables of interest, 34 variables (31.48%) had some amount of missing data. Of the 176 participants included (i.e., completed at least 90% of the items that constitute the total scores for the variables of interest), 32 (18.18%) had some amount of missing data. Of the 19,008 values in this data, 43 (0.23%) of values were missing. As such, imputing the 43 missing values would increase our sample size for modelling from 144 to 176.

## Imputation Procedure

Little's MCAR test (Little, 1988) suggested that the missing values were missing at random [$X^2$(3199) = 3280.84, $p$ = 0.15]. The Estimation Maximization procedure on SPSS was utilized to impute the missing values. Specifically, values were imputed at the subscale level. For example, values in the trust-violation subscale of the MIOS were imputed separately than values in the shame-related subscale of the MIOS. Average scores were compared between the complete case and imputed samples (Table S1).

**Table S1**

*Average Scores and Standard Deviations for Complete Case Sample and Imputed Sample*

| | **Complete Case Sample (N=144)** | | **Imputed Sample (N=176)** | |
|---|---|---|---|---|
| **Construct** | **M** | **SD** | **M** | **SD** |
| MIOS Total | 22.22 | 10.26 | 22.35 | 10.10 |
| ACES Total | 2.54 | 2.35 | 2.49 | 2.33 |
| AUDIT Total | 4.02 | 4.80 | 3.97 | 4.83 |
| CUDIT Total | 1.94 | 3.97 | 1.85 | 3.92 |
| SCS Total | 2.71 | 0.71 | 2.70 | 0.70 |
| MSPSS Total | 5.15 | 1.29 | 5.17 | 1.32 |
| SPOS Total | 52.06 | 25.18 | 52.81 | 25.02 |
| FACIT-Sp Total | 2.01 | 0.82 | 1.98 | 0.83 |
